# Oral Therapeutics Post Menopausal Osteoporosis

**DOI:** 10.7759/cureus.42870

**Published:** 2023-08-02

**Authors:** Ryan J Schroeder, Julia Staszkiewicz, Collyn O'Quin, Brandon Carroll, Nicolette Doan, Sagar Patel, Shahab Ahmadzadeh, Anusha Kallurkar, Omar Viswanath, Giustino Varrassi, Sahar Shekoohi, Alan D Kaye

**Affiliations:** 1 Medicine, Louisiana State University Health Sciences Center New Orleans, New Orleans, USA; 2 Anesthesiology, Louisiana State University Health Sciences Center, Shreveport, USA; 3 School of Medicine, Louisiana State University Health Sciences Center, Shreveport, USA; 4 Anaesthesiology, Louisiana State University Health Sciences Center, Shreveport, USA; 5 Pain Management, Valley Pain Consultants - Envision Physician Services, Phoenix, USA; 6 Pain Medicine, Paolo Procacci Foundation, Rome, ITA

**Keywords:** selective estrogen receptor modulators, hormonal therapy, bisphosphonates, oral therapeutics, fracture risk, osteoporosis, post-menopausal

## Abstract

Osteoporosis affects a significant number of postmenopausal women in the United States. Screening is performed using clinical assessments and bone mineral density scans via dual x-ray absorptiometry. Oral therapy is indicated to prevent pathologic fractures in those deemed at increased risk following screening. Bisphosphonates including alendronate, ibandronate, and risedronate are currently first-line oral therapeutics in fracture prevention following the diagnosis of osteoporosis. Hormonal therapies include estrogen-containing therapies, selective estrogen receptor modulators, and other compounds that mimic the effects of estrogen such as tibolone. Lifestyle modifications such as supplementation and physical activity may also contribute to the prevention of osteoporosis and are used as adjuncts to therapy following diagnosis. These therapeutics are limited primarily by their adverse effects. Treatment regimens should be tailored based on significant risk factors demonstrated by patients, adverse effects, and clinical response to treatment. The most severe risk factors relevant to pharmacological selection involve hormone replacement therapies, where concern for venous thrombosis, coronary artery disease, breast, and uterine cancer exist. Bisphosphonates are most commonly associated with gastrointestinal discomfort which may be mitigated with proper administration. Although adverse effects exist, these medications have proven to be efficacious in the prevention of vertebral and non-vertebral fractures in post-menopausal women. Fracture risk should be weighed against the risk of adverse events associated with each of the regimens, with clinical judgment dictating the treatment approach centered around patient goals and experiences.

## Introduction and background

Osteoporosis affects over 12 million individuals in the United States, with post-menopausal women being at increased risk [1]. There exists a multi-factorial interaction in aging women, including estrogen decline, increasingly sedentary lifestyle, chronic inflammation, and other related factors leading to the aberrant activation of osteoclasts and increased bone resorption [2]. Although osteoporosis may remain clinically silent, there is an increased fracture risk associated with osteoporotic bone resorption. Vertebral fractures are the most common manifestation with associated complications and subsequent fracture risk warranting early treatment [3]. Clinical screening for osteoporosis is recommended for post-menopausal women at age 50, with bone mineral density (BMD) measurements via dual energy x-ray absorptiometry (DXA) beginning at the age of 65. BMD assessments for those younger than 65 are indicated when an increased risk of fracture is present [1,4]. T-scores are assigned based on DXA results and, combined with fracture history and fracture risk as indicated by the US-Adapted FRAX model, are used to diagnose osteoporosis and as indications for pharmacological intervention. Intervention is recommended with a T-score of -2.5 or less at the femoral neck, hip, or lumbar spine or with a T-Score between -2.5 and -1 when a positive history of fragility fracture, current fracture, or increased fracture risk indicated by FRAX score is present [5]. Bisphosphonates are the current gold standard for oral therapeutics in osteoporosis, with consideration of other therapies generally reserved for cases of intolerance [6]. Other oral therapies considered preventative in osteoporosis during the post-menopausal period include hormone replacement therapy (HRT) and selective estrogen receptor modulators (SERM). The SERM raloxifene is not only preventative but often used in therapeutic regimens following diagnosis. Oral supplements and lifestyle modifications are also recommended. These methods are minimally invasive, low risk, and contain general health benefits outside osteoporosis prevention. Recommendations for weight-bearing exercise, calcium and vitamin D supplementation, and other such minor changes limiting caffeine, alcohol, and tobacco consumption can play a role in both prevention and treatment in select populations [3,4]. Tibolone is another oral preventative agent with a mechanism similar to that of estrogen-based therapies, although it is not currently available in the US [7]. The primary goal of all interventions in osteoporosis is to prevent further increases in fracture risk. It is important to note and counsel patients that intervention does not cure the disease but can be effective at significantly reducing fracture risk when appropriately implemented, thus reducing the overall morbidity of those patients diagnosed. The present review evaluates the mechanism of action, clinical indications, efficacy, and other considerations in oral pharmaceuticals indicated in post-menopausal osteoporosis, with a brief discussion of supplementation and lifestyle modifications.

## Review

Supplementation and behavioral intervention

Calcium and Vitamin D Supplementation 

Calcium supplementation has previously been used to prevent osteoporotic fractures, with data showing modest positive effects on bone density and reductions in fracture rates [8,9]. However, more recent studies suggest that the benefits associated with calcium supplementation depend on the degree of deficiency. In populations without significant deficiencies, supplementation has not shown a significant positive effect on outcomes, both with and without vitamin D supplementation [10-12]. It has also been shown that vitamin D is unlikely to have substantial benefits without concomitant calcium supplementation [13-15]. In light of this, the United States Preventive Services Task Force (USPSTF) recommends against daily vitamin D and calcium supplementation in community-dwelling, postmenopausal women specifically for preventing fractures, assuming that adequate intakes are established in this population [16]. However, sufficient intakes of calcium and vitamin D are recommended by the Endocrine Society and The North American Menopause Society for patients undergoing oral therapy for osteoporosis. Proper intake mitigates the risk of hypocalcemia resulting from treatment due to increased demands seen with anabolic agents and decreased mobilization seen with anti-resorptive [5,17]. It should be noted that supplementation is not without adverse effects. Calcium and vitamin D have been linked to a significant increase in urinary tract stones and a potential link between calcium supplementation and adverse cardiac events has also been demonstrated [18-20].

Nutrition and Lifestyle Modification

When treating postmenopausal osteoporosis, nutritional intake extends beyond calcium and vitamin D to mitigate risk. A significant positive association has been demonstrated between dairy product consumption and bone mineral density [21]. High protein intake has also improved lean body mass, muscle size, and bone mineral density in postmenopausal women [22]. This increase in dietary protein has also been associated with a reduced incidence of hip fractures in postmenopausal women, perhaps for the reasons stated above, as fall risk is a significant contributing factor to fracture [23]. Probiotics have also been shown to positively impact women with osteoporosis, with decreases in bone resorption leading to increases in bone mineral density [24]. Other notable supplements include magnesium, vitamin K, and phytoestrogens, although there lacks significant evidence of their benefits in most populations [4]. Physical activity, weight-bearing exercise, and fall prevention are other considerations when evaluating patients at elevated fracture risk. In postmenopausal women, simply walking has been shown to significantly benefit bone mineral density at the femoral neck [25]. A study on fall prevention demonstrated a 39% reduction in falls in older adults when training more than three hours/week [26]. Swimming is a viable alternative in select cases, as it has been shown to increase bone mineral density in postmenopausal women with lower joint impact than walking [27]. The USPSTF has issued a B recommendation for exercise interventions in preventing falls in community-dwelling individuals at an increased risk of falls, such as postmenopausal women [28].

Bisphosphonates

Mechanism of Action 

Bisphosphonates exert their therapeutic effect via inhibition of the enzyme farnesyl pyrophosphate synthase in osteoclasts [29]. Although this enzyme and subsequent cascade are utilized in various cell types, bisphosphonates exert selectivity by binding to bone minerals, exposing osteoclasts to high drug concentrations [30]. The result is a relatively specific attenuation of osteoclast activation leading to a net decrease in the resorption of bone surface area [29]. 

Clinical Indications

There are currently three widely available and recommended oral bisphosphonates for preventing and treating postmenopausal osteoporosis. These include alendronate, ibandronate, and risedronate [4,5]. All three have been shown to significantly reduce fracture rates in postmenopausal women when compared to placebo and are considered first-line oral therapy [5]. Alendronate and risedronate have been shown to reduce hip, vertebral, and non-vertebral fractures, with risk reduction being more pronounced in vertebral than non-vertebral fractures [4]. Ibandronate has been shown to exhibit the slightest effect on vertebral fractures and seems to have no observable impact on non-vertebral fracture risk [31-34]. 

Adverse Effects, Drug Interactions, and Contraindications

Bisphosphonates have been linked to upper gastrointestinal adverse effects such as nausea, vomiting, pain, and dyspepsia. Despite observed associations in small clinical studies, larger trials and meta-analyses struggle to find a strong association between bisphosphonates and gastrointestinal (GI) adverse events compared to placebo [35-38]. A 2020 meta-analysis showed a substantial number of events occurred within the esophagus. With other evidence pointing towards GI events being attributed to improper drug administration when taken orally, proper administration protocols become paramount [35,39]. Prevention of gastrointestinal events in bisphosphonate therapy relies upon appropriate dosing and administration [40,41]. Bisphosphonates must be taken fasted with water, and the patient should be instructed to remain fasted and upright for two hours following administration to maximize absorption and reduce the risk of adverse effects. Newer formulations of bisphosphonates are also available to reduce GI events, specifically in individuals with pre-existing GI conditions [42]. Other rare, more severe adverse effects of bisphosphonate therapy include atypical femoral fractures and jaw osteonecrosis [42,43]. Atypical femoral fractures exhibit a duration-dependent risk increase throughout bisphosphonate-based regimens [44-47]. These risks have led to the recommendation of "drug holidays." Bisphosphonates retain their therapeutic effect for up to five years after discontinuation of therapy, indicating that temporary cessation of treatment may be used to reduce the risk of adverse events while maintaining osteoporotic fracture risk reduction [46,48-50]. The American Society for Bone and Mineral Research recommends that those at low fracture risk may consider a two to three-year drug holiday. However, in patients with a higher risk of fracture, the relatively low risk of atypical femoral fracture or osteonecrosis of the jaw may be outweighed, and therapy is maintained [46].

Selective estrogen receptor modulators

Selective Estrogen Receptor Modulators (SERMS) are a class of medications with both agonistic and antagonistic effects on estrogen receptors. SERMs exhibit tissue-specific agonist/antagonist activity by interacting with differing subunits of the estrogen receptor, α, and β [51]. The estrogen receptor alpha subunit (ER-α) is predominately expressed in tissues including the uterus, prostate, ovary, testes, bone, breast, and liver. In contrast, the estrogen receptor beta (ER-ß) is expressed in tissues such as the colon, bone marrow, salivary gland, and vascular endothelium [52]. When estrogens and SERMs bind to ER-α, gene transcription is initiated. Coupling to ER-ß does not exert transcriptional activity and demonstrates an antagonistic effect on ER-α signaling [53,54]. These therapies are particularly useful in treating and preventing estrogen-dependent conditions where their receptor selectivity can be leveraged in patients at increased risk of breast or uterine cancer, such as those with a positive family history. Concerning osteoporosis, SERMs exhibit agonistic effects on estrogen receptors related to bone homeostasis while antagonizing those mediating proliferative effects in uterine and breast tissue [55]. Drugs of this class that have proven helpful in osteoporosis include raloxifene and basedoxifene. Raloxifene is approved in the US to prevent and treat postmenopausal osteoporosis [56]. Bazedoxifene is supported to avoid postmenopausal osteoporosis in combination with conjugated estrogen [57]. 

Raloxifene

Mechanism of Action

Raloxifene's estrogenic effects on bone reduce resorption and increase bone mineral density [58]. The drug exerts its effects via TGF-ß3 and IL-6 gene expression regulation and estrogen agonism in pre-osteoclasts. The combined effect is a reduction in net bone resorption [59]. Raloxifene also antagonizes estrogen-mediated effects in mammary and uterine, inhibiting estradiol-dependent proliferation in human mammary tumor cells in vitro. Although the mechanism of action is not fully understood, it is speculated that structural differences between the SERM-ER complex and the Estrogen-ER complex play a role. The existence of two ERs may contribute to its tissue specificity [58,60,61].

Clinical Indications

Raloxifene is Food and Drug Administration (FDA)-approved as a monotherapy in treating and preventing post-menopausal osteoporosis. Raloxifene has been shown to reduce vertebral fracture risk in large placebo-controlled trials. However, the drug has yet to be directly compared to bisphosphonates. Additionally, raloxifene has not significantly reduced non-vertebral and hip fracture risk. Thus, its clinical utility lies predominately in patients with osteoporosis with significant spine BMD deficits [62]. Raloxifene is also approved for invasive breast cancer risk reduction and treatment. Evidence suggests it may mitigate post-menopausal symptoms in women, leading to its specific recommendation in patients with osteoporosis and a high risk of invasive breast cancer, assuming the contraindications discussed below are absent [53,62]. A randomized controlled trial on raloxifene in breast cancer patients demonstrated significant reductions in new vertebral fractures with a concurrent decrease in the ER+ breast cancer occurrence compared to placebo [63,64]. These results were supported in the multiple outcomes of raloxifene evaluation (MORE_ trial, where raloxifene showed efficacy in treating osteoporosis and hormone-responsive breast cancer without an increased risk of tissue proliferative side effects [64].

Adverse Effects and Contraindications

Significant adverse effects of raloxifene include an increased risk of venous thromboembolism (VTE), hot flashes, stroke, lower extremity edema, and lower extremity cramps [65]. In the continuing outcomes relevant to evista (CORE)-MORE trial, a long-term continuation of the MORE study, an increased risk of venous thromboembolism was observed compared to the placebo. However, the incidence of hot flashes, extremity cramping, and peripheral edema was not statistically significant between groups [66,67]. Cardiovascular effects were also investigated in the CORE-MORE trial, with raloxifene having no considerable increase in the incidence of coronary events [67]. The raloxifene use for the heart (RUTH) trial confirmed these results with raloxifene demonstrating no increased risk of coronary heart disease; however, death from stroke was increased in select populations [68]. These populations were later determined to have increased Framingham Stroke Risk Scores (FSRS) independent of raloxifene use [69]. Combining evidence from the MORE, CORE, and RUTH trials, raloxifene has been significantly associated with hot flashes compared to placebo, with hot flashes being dose-dependent [62].

Based on these findings, raloxifene is not recommended in patients with a history of deep vein thrombosis, retinal vein thrombosis, pulmonary embolism, or those with typical risk factors for VTE. Additionally, raloxifene prescription should be based on clinical judgment with caution in women with a high risk of coronary heart disease or stroke, as defined by an FSRS score > 13. Raloxifene should also be avoided or dose adjusted in women with vasomotor symptoms such as hot flashes [62]. The actual clinical utility of raloxifene lies within the population of patients that cannot tolerate bisphosphonates and those diagnosed or at increased risk of hormone-responsive breast cancer. However, most medical recommendation agencies leave this decision to clinical judgment and individual evaluation. 

Bazedoxifene

Bazedoxifene is approved by the FDA for post-menopausal osteoporosis prevention when combined with conjugated estrogens (CE/BZA) [70]. This combination therapy has been shown to reduce osteoporotic fracture risk and vasomotor symptoms associated with menopause in women with a uterus [71]. Two large phase III clinical trials have shown bazedoxifene to be as productive as raloxifene in increasing vertebral BMD while improving femoral neck and femoral trochanter BMD compared to placebo [72,73]. In another two-year investigation, bazedoxifene demonstrated significant improvements in vertebral BMD at all doses compared to placebo. Additionally, bazedoxifene significantly reduced vertebral and non-vertebral fractures compared to placebo [72,74].

Mechanism of Action

Bazedoxifene favors postmenopausal osteoporosis, lipid levels, breast, and uterine tissue [75]. Bazedoxifene exhibits antagonistic activity in the breast and uterine tissues, impeding the proliferative effects of estrogen in ER-positive cells of these tissues. Bazedoxifene acts as an estrogen agonist in lipid metabolism, effectively lowering LDL and total cholesterol levels. In bone, inhibition of bone resorption and elevations in bone mineral density give rise to its utility in postmenopausal osteoporosis [76].

Clinical Indications

Indications for the use of bazedoxifene/conjugated estrogen combination therapy include treating patients with moderate to severe vasomotor symptoms and prevention/treatment of osteoporosis in postmenopausal women. Additionally, this combination treatment should be limited to those at high risk for osteoporosis [77].

Adverse Effects and Contraindications

CE/BZA therapy is associated with adverse events, including muscle spasms, dizziness, nausea, abdominal pain, and hot flashes, with the latter three most commonly leading to discontinuation. The same warnings and precautions as other estrogen-containing medications exist for this regimen. Patients should be monitored for thromboembolic events and instructed to report abnormal vaginal bleeding to their healthcare provider [77]. Bazedoxifene has similar contraindications to other SERMs previously discussed, including an increased risk of venous thromboembolism. Although not expressly contraindicated, bazedoxifene is not recommended for treating those with past thromboembolic events, as extreme caution must be demonstrated [71].

Hormonal therapy

The use of hormone replacement therapy as a first-line treatment for osteoporosis in perimenopausal and post-menopausal women has been largely debated. The Woman's Health Initiative (WHI) conducted a study to gauge the health risk-to-benefit ratio of hormone replacement therapy for the primary treatment of osteoporosis in postmenopausal women. It determined that the health risk associated with hormonal treatment outweighed the benefits [78]. These risks include an increased incidence of breast cancer, coronary heart disease, and venous thromboembolism. Therefore, hormonal therapy as primary osteoporosis treatment is not widely utilized. 

Mechanism of Action

Declining estrogen levels in perimenopausal and postmenopausal women lead to a rapid loss of bone mineral density and thus predispose to increased fractures [79]. Estrogen therapy and hormone therapy (estrogen plus progesterone combination) aim to stabilize bone remodeling to pre-menopausal equilibrium states [51]. While estrogen plays a role in managing calcium absorption in the intestines and kidneys, more recent research has shown that estrogen's primary role in osteoporosis pathology is the loss of homeostasis between osteoclasts and osteoblasts related to lack of estrogen [51]. 

Estrogen stimulates osteoclast apoptosis while inhibiting osteocyte and osteoblast apoptosis [80]. Osteoclast stimulation is mediated by estrogen deficiency-related increased levels of TNF-α and IL-1. These cytokines increase the levels of receptor activator of nuclear factor Kappa-B ligand (RANKL), a ligand that increases osteoclast differentiation and activity. The result as estrogen levels decline is a shift in the balance between osteoblasts and osteoclasts, leading to net bone resorption [80]. The primary goal of hormonal therapy is to re-establish this equilibrium and prevent increased bone resorption associated with declining estrogen levels in postmenopausal women. 

Estrogen and Progestogen Treatment

Women without a uterus receive 0.625mg estrogen replacement alone, such as Premarin, with women with a uterus receiving a combination of 0.625mg estrogen and 2.5mg progesterone, such as Prempro, as therapy for osteoporosis [78]. Before the WHI study results, a cohort study published in 1995 examined estrogen replacement therapy in relation to fractures in older women. It was demonstrated that initiation of hormone therapy within five years of menopause was linked to decreased incidence of the hip, wrist, and all non-spinal fractures when compared to women not placed on hormone replacement therapy [81]. One placebo-controlled trial observing the effects of low-dose estrogen replacement therapy used 0.014mg of estrogen-only hormone replacement therapy. The result was a 2.6% bone mineral density increase in the lumbar region compared to 0.6% in the placebo group. There was also a 0.4% increase in hip bone mineral density as opposed to a 0.8% decrease in the placebo group [80]. This serves as a quantification method for the effects of estrogen therapy as a treatment for postmenopausal osteoporosis.

Adverse Effects

While the efficacy of hormone replacement therapy has been shown through multiple studies, the Women’s Health Initiative questions whether the risk exceeds the benefits [82]. The WHI conducted a randomized controlled primary prevention trial on over 16,000 women throughout 40 clinical centers across the U.S. over five years. This large-scale study showed that women on estrogen plus progesterone therapy had a 29% increased risk for coronary heart disease, 41% increased risk for stroke, and twice as likely to experience a venous thromboembolic event compared to placebo [82]. The trial also showed a 26% increase in breast cancer. The adverse effects observed resulted in early termination of the study and the recommendation for other treatment modalities in postmenopausal osteoporosis [78].

Tibolone

Mechanism of Action

An analog of the progestin norethynodrel, tibolone is a steroid with estrogenic, progestogenic, and androgenic properties following intestinal conversion into metabolites that exert tissue-specific activity [83]. Additionally, it can inhibit steroid-metabolizing enzymes and induce enzymes involved in nullifying estrogenic activity [7]. These dual pathways of tibolone exert clinically beneficial effects on bone, vaginal, and central nervous system tissues while avoiding stimulation of endometrial and breast tissue [84]. Regarding postmenopausal osteoporosis, hormone replacement therapy such as tibolone prevents accelerated bone turnover and loss [85].

Clinical Indications 

Tibolone has been utilized as a low-risk hormone replacement alternative to estrogen therapy in postmenopausal women. Tibolone has been shown to positively affect bone mineral density compared to estrogen therapy, with fewer adverse effects [86]. Additionally, tibolone significantly reduces non-vertebral and vertebral fractures in postmenopausal women [40]. Tibolone may be preferred over estradiol-based HRT in postmenopausal women with residual endometriosis, as demonstrated in a 2017 review; however, limitations in sample size merit further investigation [87,88]. Both the North American Menopause Society and the Endocrine Society recommend using tibolone to prevent osteoporosis [5,17]. Despite these recommendations, tibolone is not currently available in the United States. However, it is now available in over 70 countries [89,90].

Adverse effects, Contraindications, and Drug Interactions

Although tibolone has been shown to have decreased stimulation of both breast and endometrial tissues when compared to other hormonal therapies, similar adverse effects remain. While not associated with increased endometrial cancer, tibolone was linked to breast cancer recurrence and is therefore contraindicated following breast cancer treatment [90,91]. Other potentially serious adverse effects include an increased risk of stroke for older osteoporotic women, although the quality of evidence warrants further investigation [90,92]. The most common adverse effects of tibolone include leukorrhea, abdominal pain, weight increase, breast pain, and vaginal bleeding [93]. However, evidence indicates that vaginal bleeding and breast pain are more familiar with continuous estrogen/progestogen therapy than with tibolone [94]. Additionally, tibolone does not affect liver or renal function [93]. With these potential risks, tibolone should be given similar consideration as those with hormonal replacement therapy in individuals at increased risk for breast cancer and stroke until further evidence further elucidates the adverse effect profile of this therapeutic. 

Oral therapeutics for post-menopausal osteoporosis

Results from a 2019 large-scale meta-analysis of 107 trials assessing fracture risk following oral therapeutic regimens in 193,987 post-menopausal women can be seen in Table 1 [40]. Statistically significant reductions in relative fracture risk compared to placebo were seen at both vertebral and non-vertebral locations in the bisphosphonates alendronate and risedronate. Ibendronate also saw a decline in vertebral fracture risk how; however-vertebral fracture risk did not reach significance, with SERMs raloxifene and bazedoxifene exhibiting similar results. Hormonal replacement therapy consists of a combination of estrogen and progesterone therapy. Both HRT and Tibilone reduced both vertebral and non-vertebral fracture relative risks. Vitamin D supplementation alone showed a statistically significant decrease in non-vertebral fracture risk, while a combination of vitamin D and calcium did not in this population.

**Table 1:** Oral therapeutics for Post-Menopausal Osteoporosis [40] *Statistically significant, CI: confidence interval, RR: relative risk compared to placebo, HRT: hormone replacement therapy

Oral Therapeutic Agent	RR Non-vertebral Fracture	95% CI	RR vertebral fracture	95% CI
Alendronate	0.84	0.74-0.94*	0.57	0.45-0.71*
Ibandronate	1.06	0.83-1.36	0.67	0.48-0.93*
Risedronate	0.78	0.68-0.89*	0.61	0.48-0.78*
HRT	0.78	0.68-0.89*	0.65	0.46 – 0.98*
Raloxifene	0.94	0.85 – 1.05	0.59	0.46 – 0.76*
Bazedoxifene	0.90	0.72 – 1.11	0.61	0.41 – 0.90*
Tibolone	0.73	0.58-0.94*	0.56	0.36 – 0.87*
Vit. D	0.44	0.23 – 0.85*	0.85	0.46 – 1.59
Vit. D + Calcium	0.93	0.85 – 1.01	0.88	0.61 – 1.27

## Conclusions

The goal of oral therapy in post-menopausal osteoporosis is a reduction in fracture risk, with bisphosphonates as the current first-line for prevention of both vertebral and non-vertebral fractures. Hormonal-based therapies, including estrogen regimens, selective estrogen receptor modulators, and tibolone, may be used with caution in those with a significant personal or family history of breast or uterine cancer, venous thrombosis, cardiovascular disease, and stroke. Once a treatment approach has been selected, counseling on lifestyle modifications and supplementation may play an adjunctive role in select populations. Routine follow-ups to assess adverse effects and treatment success are needed to tailor care to individual patients' continual needs and goals.
